# Techno-Economic Assessment and Sensitivity Analysis of Glycerol Valorization to Biofuel Additives via Esterification

**DOI:** 10.1021/acs.iecr.3c00964

**Published:** 2023-05-31

**Authors:** Krutarth Pandit, Callum Jeffrey, John Keogh, Manishkumar S. Tiwari, Nancy Artioli, Haresh G. Manyar

**Affiliations:** † School of Chemistry and Chemical Engineering, Queen’s University Belfast, David-Keir Building, Stranmillis Road, Belfast BT9 5AG, U.K.; ‡ Department of Chemical Engineering, Mukesh Patel School of Technology Management and Engineering, SVKM’s NMIMS University, Mumbai 400056, Maharashtra, India; § Department of Civil, Environmental, Architectural Engineering and Mathematics, University of Brescia, Via Branze, 43, 25123 Brescia, Italy

## Abstract

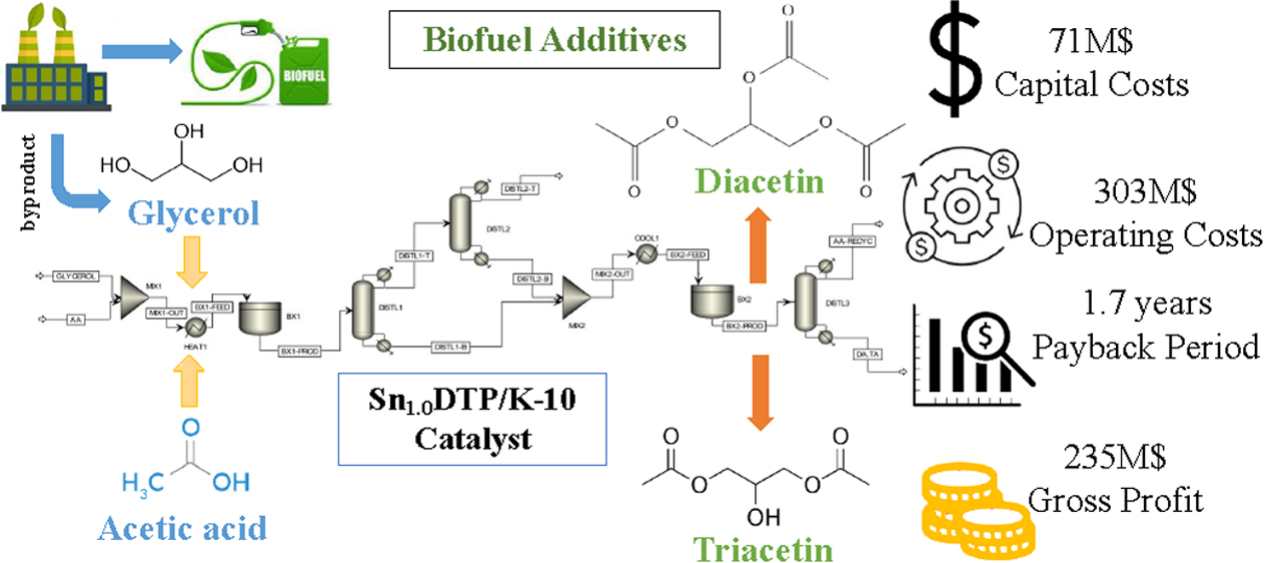

Glycerol is a valuable feedstock, produced in biorefineries as a byproduct of biodiesel production. Esterification of glycerol with acetic acid yields a mixture of mono-, di-, and triacetins. The acetins are commercially important value-added products with a wide range of industrial applications as fuel additives and fine chemicals. Esterification of glycerol to acetins substantially increases the environmental sustainability and economic viability of the biorefinery concept. Among the acetins, diacetin (DA) and triacetin (TA) are considered high-energy-density fuel additives. Herein, we have studied the economic feasibility of a facility producing DA and TA by a two-stage process using 100,000 tons of glycerol per year using Aspen Plus. The capital costs were estimated by Aspen Process Economic Analyzer software. The analysis indicates that the capital costs are 71 M$, while the operating costs are 303 M$/year. The gross profit is 60.5 M$/year, while the NPV of the project is 235 M$ with a payback period of 1.7 years. Sensitivity analysis has indicated that the product price has the most impact on the NPV.

## 1 Introduction

There has been an increase in global energy demand due to the growing population, industrialization, and our need for a better quality of life. This necessitates the efficient production of renewable energy due to the limited availability of fossil fuels and the climate and environmental issues associated with the use of these fossil fuels.^1,2^ With the transportation sector considered as one of the most difficult areas to decarbonize, the increased penetration of “green”, sustainable fuels is imperative.^3^ Biodiesel has been recognized as a potential substitute to alleviate the current global dependence on petroleum-derived fuels, with several key advantages including its renewable, nontoxic, and biodegradable nature.^4^ While capable of significantly diminishing both greenhouse gas emissions and the release of harmful particulates associated with respiratory health effects, biodiesel further provides numerous logistical advantages over alternative “green” fuels such as bioethanol, biomethane, and hydrogen.^5^ Such advantages are chiefly attributed to its structural similarity to mineral diesel, enabling its direct employment within current infrastructure for transportation, storage, and handling, as well as its direct utilization within conventional compression-ignition engines without onerous adjustments.^6^

Despite this huge potential, the viability of biodiesel to displace petroleum-derived fuels is currently hindered by its production route, involving the transesterification of renewable triglycerides, such as vegetable oils and animal fats, in the presence of methanol. While capable of producing the desired fatty acid methyl esters (FAME), more commonly known as biodiesel, it adversely generates glycerol as a byproduct in significant quantities, accounting for 10 wt % of biodiesel manufacture.^7^ Depending on the application in pharmaceutical, cosmetic, and food sectors, the crude glycerol can be purified into refined glycerol, using a variety of purification techniques like distillation, membrane separation, ion exchange, and solvent extraction by acidification and neutralization. The price of the refined glycerol is 0.8–1.2 $/kg, 5–10 times more than the price of the crude glycerol.^8^ With purification processes rendered economically infeasible, a sustainable valorization pathway to upgrade the surplus glycerol is imperative in enhancing the viability of biodiesel while mitigating the potential for glycerol to become an environmental pollutant. With a forecasted biodiesel production volume of 60 billion liters in 2025, approximately 6 billion liters of glycerol will be coproduced.^9^ While glycerol is a highly functional molecule with favorable physicochemical properties, enabling its use as a feedstock raw material for the synthesis of over a thousand fine chemicals, glycerol markets are currently saturated, incapable of handling the significant surplus volumes generated.^10,11^

**Figure 1:**
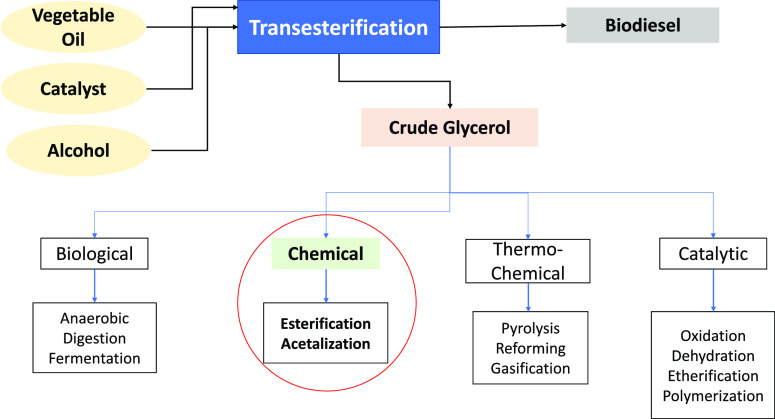
Production of biodiesel and valorization routes of crude glycerol.

Furthermore, with increasing biodiesel production resulting in the declining cost of crude glycerol, the development of an effective valorization pathway presents a very lucrative opportunity for the generation of value-added products, simultaneously promoting a circular economy and thus enhancing the viability of both the biorefinery concept and the oleochemical market (Figure 1).^12−14^ Recently, Calero et al. have investigated “greener” biodiesel manufacturing routes, whereby the coproduction of the glycerol byproduct is mitigated; such studies to date have been performed at the lab scale only and thus do not present a feasible option for commercialization.^15^ The discovery of an optimized and sustainable procedure that can effectively utilize crude glycerol generated from biodiesel manufacture will have a profound impact on the viability of the fuel in decoupling the petroleum dependence within the transportation sector.^16^

**Figure 2:**
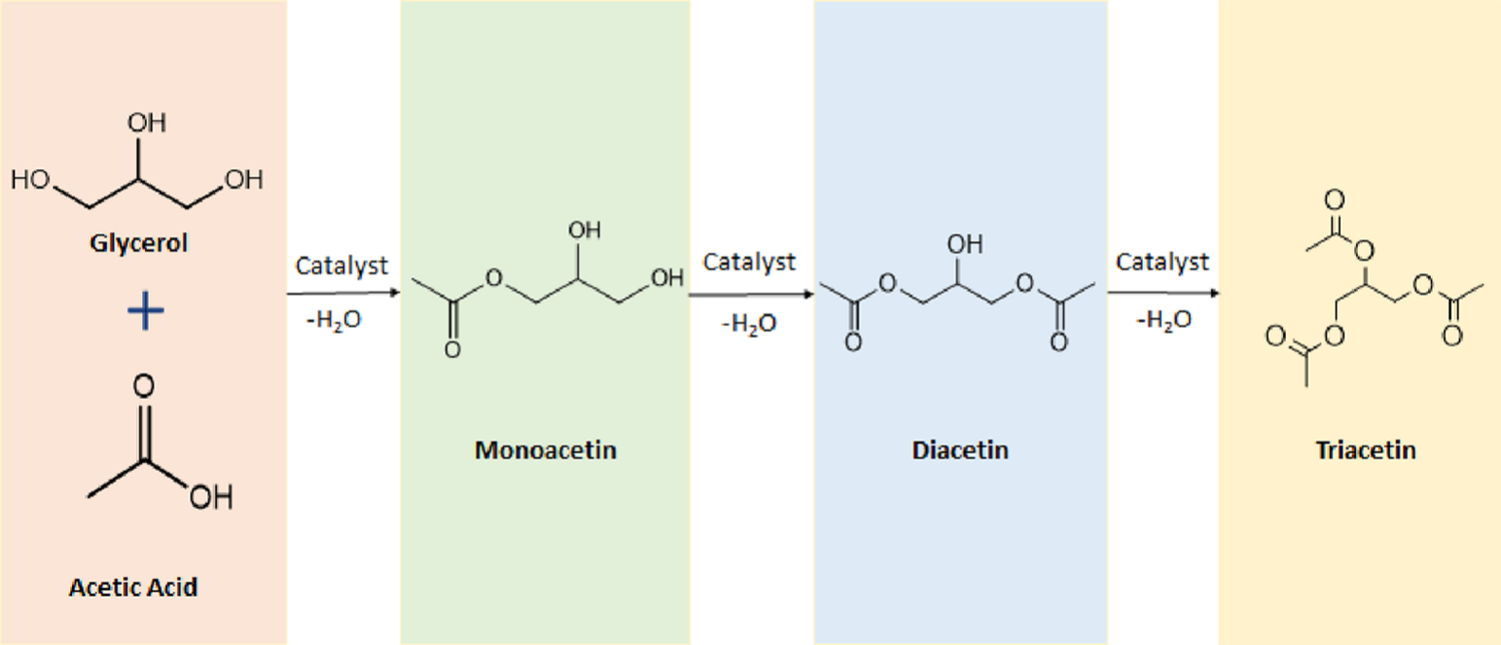
Reaction scheme for the production of acetins from the esterification of glycerol with acetic acid.^13^

The esterification of glycerol to acetins has been identified as a promising, sustainable pathway to effectively valorize surplus glycerol generated from biodiesel manufacture. The acetylation reaction can employ either acetic acid or acetic anhydride as the acetylating agent. While the use of acetic anhydride is thermodynamically favored over the application of acetic acid and can produce the desired acetins within lower temperature ranges, its much higher cost and potentially explosive nature render its use infeasible for commercialization.^17,18^ Furthermore, the acetylation of glycerol in the presence of acetic anhydride is extremely fast and as such is difficult to control.^19^ Current research developments thus predominantly consider the optimization of the esterification reaction utilizing acetic acid. The esterification process consists of three consecutive equilibrium reactions, as shown in the reaction scheme in Figure 2.

**Figure 3:**
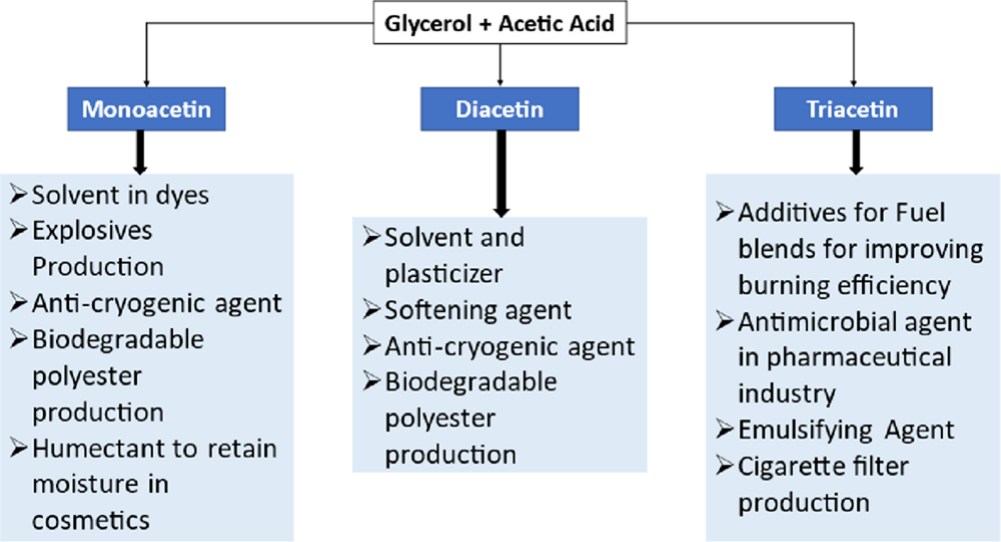
Applications of mono-, di-, and triacetin.

Acetins are extremely versatile molecules, which can be employed within a range of applications, as displayed in Figure 3. As such, they are considered extremely valuable commercial products.^20,21^ Of the multitude of products that can be obtained from the defined acetin species, blends of the di- and trisubstituted esters, diacetin and triacetin, can function as effective biofuel additives.^22^ The esterification of crude glycerol to biofuel additives operates as a double-edged sword, increasing both the economic and environmental viability of biodiesel manufacture while simultaneously improving fuel quality with respect to low-temperature flow properties, octane number, and reduced CO emissions.^13,23^ Furthermore, in recent years, the demand for such esters has experienced a continual annual growth rate of between 5 and 10%, with future demands expected to remain strong.^18^ As such, the direct valorization of crude glycerol to high-content triacetin fuel additives presents an extremely attractive production route, capable of generating both an in-demand commodity while simultaneously boosting the biodiesel market. When considering the defined esterification reaction within the scope of biofuel additive synthesis, high selectivity toward diacetin and triacetin is required, with an enhanced focus on generating the latter molecule; due to the soluble nature of monoacetin within polar media such as water, it is considered an undesired byproduct of the reaction mechanism.

In continuation of our group’s interest in environmental catalysis, the aim of this work is to provide a feasibility study with a techno-economic analysis for the valorization of crude glycerol into acetins.^24−33^ This analysis has been done on Aspen Plus for process optimization followed by the techno-economic analysis by Aspen Process Economic Analyzer (APEA). The first part consists of a preliminary process development and a preliminary conceptual flowsheet with the mass and energy transfer schemes. The second part consists of the economic assessment where further financial aspects have been covered. A discounted profit flow analysis has been done to estimate the net present value (NPV) and the payback period for the proposed project along with a sensitivity analysis showing the implications on the NPV and payback period. The study can thus quantify the feasibility of glycerol valorization to acetins via esterification with high industrial importance.

## 2 Development of the Aspen Plus Model

### 2.1 Defining Nondatabank Components 1-Monoacetin and 1,3-Diacetin

Prior to simulation, it was required to manually define both 1-monoacetin and 1,3-diacetin within the software due to both molecules being nondatabank components. Utilizing a procedure reported by Luyben and Chien^34^ and adopted by both Hung et al.^35^ and Souza et al.,^23^ both components were input within the software through defining their molecular structure and simulating their physical property parameters utilizing the built-in NIST TDE approach. It is important to note that only 1-monoacetin and 1,3-diacetin were considered within the modeling, with the isomers 2-monoacetin and 1,2-diacetin neglected. This approach was employed within the previously defined Aspen modeling studies considering the glycerol esterification reaction mechanism due to their comparatively infinitesimal quantities.

### 2.2 Physical Property Method Selection and Binary Parameter Estimation

The selection of an appropriate physical property method within Aspen Plus is essential in ensuring that the simulation yields reliable, accurate results. With respect to the defined liquid-phase esterification reaction, the application of a liquid activity coefficient property method is recommended within both the literature and the Aspen User Handbook. For such a reason, the UNIQUAC-NTH equation of state was selected.

### 2.3 Proposed Process Flowsheet

With the defined project aiming to operate as a preliminary proof of concept to assess the commercial viability of the investigated catalyst at an industrial production scale, it was desired to first model the process as a batch operation. Such an approach is common within an early process development phase, operating as an effective methodology to assist in both economic analysis and catalyst viability studies. Further, due to the kinetic modeling study considering experimental data collected during batch trials,^36^ the consideration of batch modeling utilizing such kinetics was a logical starting point. While patented continuous processes for triacetin production exist, a significant portion of the global output is manufactured via batch processing.^37^

**Figure 4:**
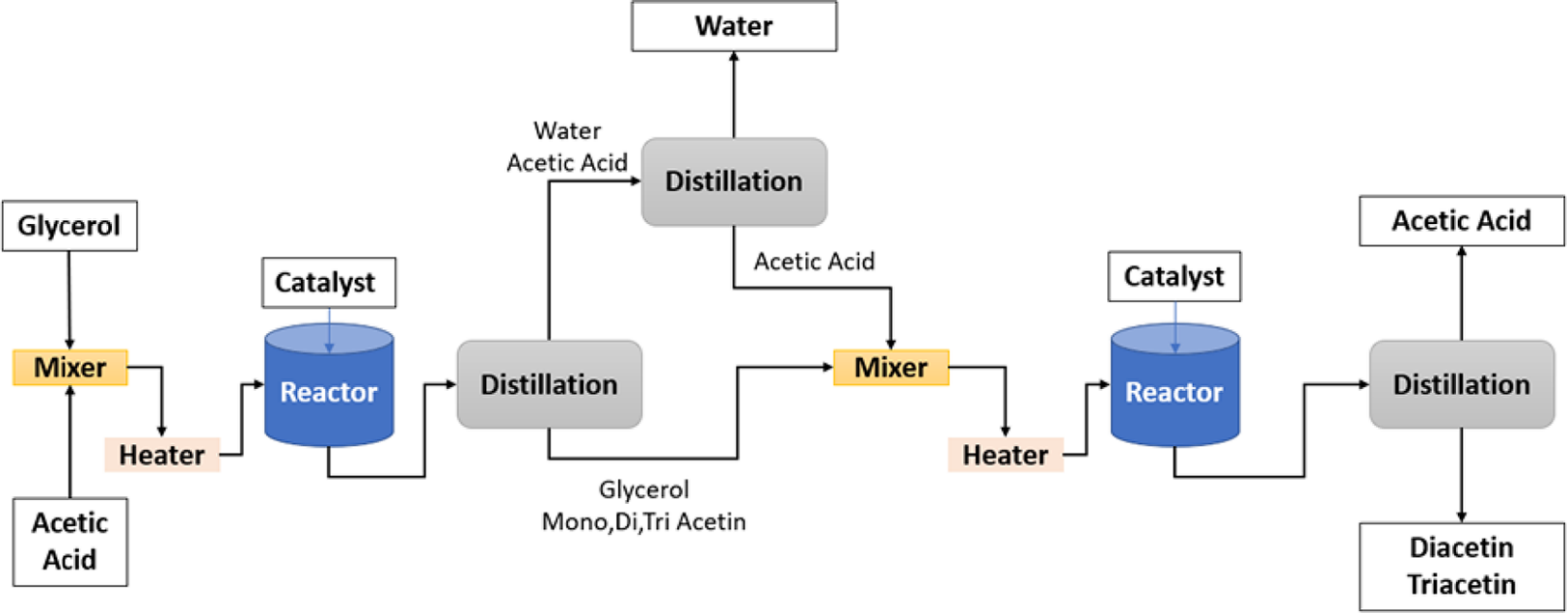
Block diagram of the proposed process.

**Figure 5:**
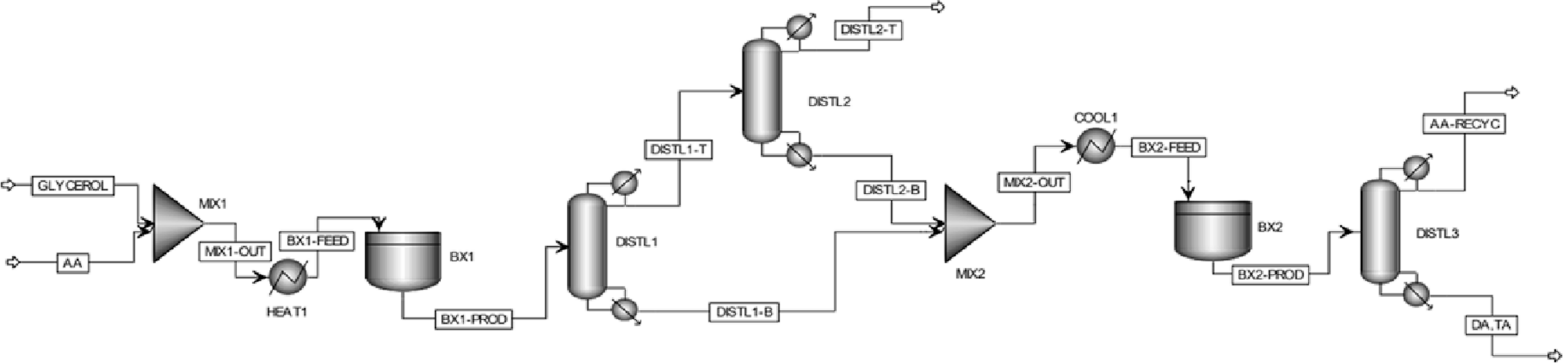
Proposed batch modeling flowsheet on Aspen Plus.

Because of the preliminary experimental work indicating an inability to achieve complete selectivity to the desired higher esters, diacetin and triacetin, within a single batch reactor stage, it was decided to first consider a two-stage reaction process. Following the first batch reactor, the product enters a distillation column, defined as “DISTL1”. The purpose of this preliminary column is to remove all water cogenerated by the esterification reactions and thus remove the inhibiting presence of water from the reaction medium, which restricts the position of equilibrium. Due to the proximity in boiling points of acetic acid and water, a secondary column, “DISTL2”, is required to effectively recover the acetic acid lost in the distillate of the primary column; such acetic acid is recovered efficiently in this column, leaving with high purity within the bottom stream where it is subsequently utilized in the second stage reaction. The distillate of the secondary distillation column consists of an essentially pure water stream, with only trace quantities of acetic acid, which can subsequently be disposed of safely, posing no threat to the environment. Due to the high acetic acid demand required to assist in driving the position of equilibrium toward the formation of the desired higher esters, an effective acetic acid recovery system is imperative from a sustainability and economic viability perspective. The distillation sequence proposed above was developed considering distillation heuristics for favorable separations and economic operations. Within the second stage batch reaction, occurring within “BX2”, the bottom stream from the primary distillation column, consisting of a mixture of acetin species only, is fed with the recovered acetic acid. Following this second phase reaction, complete selectivity to the desired higher esters (diacetin and triacetin) could be attained, with all glycerol and monoacetin effectively converted. The product stream leaving the secondary batch reactor is fed into a final distillation column, whereby the desired product could be effectively isolated within the bottom stream with high purity, with excess acetic acid recovered within the distillate stream, which can be recycled and reused in subsequent batches. The overall material balance for the two-stage process, capable of processing 100,000 tons of glycerol per year, is reported in the Supporting Information. A simplified block diagram of the proposed two-stage process can be seen in Figure 4. Subsequently, Figure 5 shows the Aspen Plus diagram of the proposed process.

### 2.4 Optimization of the Flowsheet with Respect to Process Economics

As aforementioned, the assessment of the commercial viability of the proposed catalyst at scale was to be achieved through conducting a techno-economic analysis, considering a plant with a fixed annual capacity for processing 100,000 tons of crude glycerol. To realize the full economic potential of the proposed two-stage process more accurately, it was desired to optimize all reactors and columns with respect to their economics.

**Figure 6:**
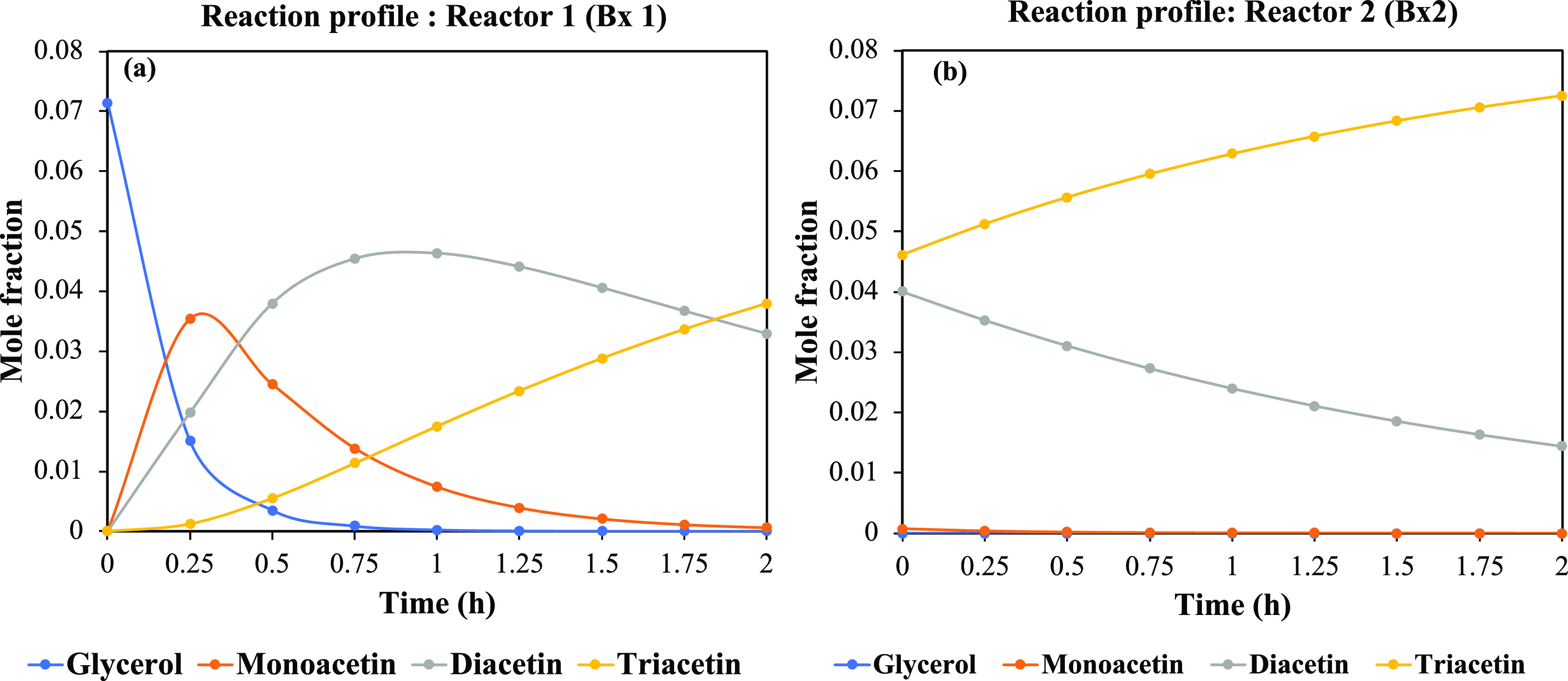
Simulated reaction profiles for both reactors.

**Figure 7:**
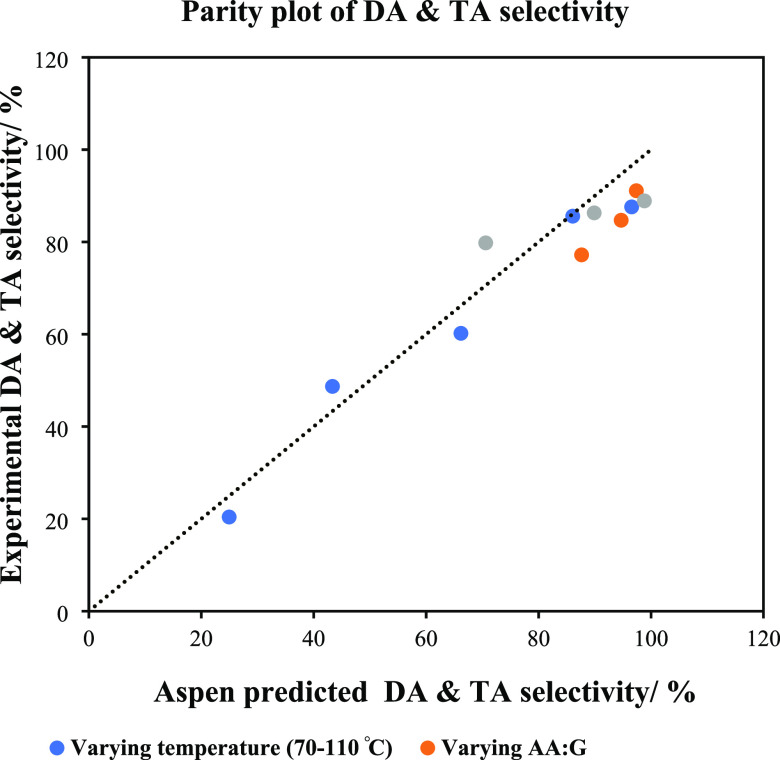
Validation of the developed Aspen model.

The complete process optimization of operating parameters along with the reaction kinetics was based on our recent study.^36^ To validate the developed Aspen model, the Aspen results were verified with the experimental findings. The Aspen model was commonly observed to marginally overpredict the combined diacetin and triacetin selectivity but to an unappreciable extent, with an average error of 6.8%. This level of inaccuracy is to be expected considering that the kinetic model was developed using experimental data at both a fixed catalyst loading and acetic acid to glycerol molar ratio. Under the same conditions, an average error of 0.6% between the experimental and predicted glycerol conversions was obtained. Because of the small margins in error upon variation of reaction conditions, the kinetic model was considered valid for the defined process optimization procedure, capable of predicting with a relatively high level of accuracy both the expected glycerol conversion and product distribution. As aforementioned, however, the kinetic model was considered only valid over the range of conditions experimentally investigated, as beyond such conditions, it was not possible to compare the model’s accuracy to experimental data. In restricting the extent to which the reaction parameters could be varied to within this range, there was confidence in the ability of the Aspen Plus model to predict realistic reaction phenomena. Figure 6 shows the simulated product profiles for both reactors. Figure 7 shows the parity plot between our recent experimental study^36^ vs the developed Aspen Plus model in this study.

Table 1 shows the optimum process parameters that were used in the Aspen flowsheet after detailed optimization of the process parameters in our recent experimental study.^36^ For an economic optimization of the distillation column, the RadFrac option was utilized as recommended by Luyben and Chien,^35^ on account of its more rigorous calculation procedure within Aspen Plus, resultantly leading to a more realistic process economics estimation. Additionally, a Langmuir–Hinshelwood–Hougen–Watson (LHHW) reaction set based on the kinetic data from our previous study was used in the flowsheet as well.^36^Table 2 shows the effects of different operating parameters on the overall process economics.

**Table 1:** Economically Optimized Process Parameters

process parameter	value
acetic acid:glycerol (wt)	13:1
temperature	110 °C
catalyst loading	13 wt %
batch time	2 h

**Table 2:** Influence of Operating Conditions on Process Economics

operating condition	influence on process economics
Higher reaction temperatures	Increased operating costs (OPEX):
	Increased utility demand to bring the reactant mixture to the desired temperature
Higher acetic acid to glycerol molar ratios within the feed stream	Increased operating costs (OPEX):
	Increased utility demand to bring the reactant mixture to the desired temperature (due to increased batch size)
	Increased feedstock demand
	Increased capital investment (CAPEX):
	Increased reactor size to accommodate larger batch charge
Higher catalyst loading	Increased operating costs (OPEX):
	Increased catalyst demand
	Increased capital investment (CAPEX):
	Increased reactor size to accommodate a larger catalyst requirement
	Note: Over the range of loadings investigated, no impact on reactor cost was observed. Subsequently, no relationship was required to be established relating an increase in catalyst weight to an increase in reactor cost.

The objective function for the profit of the project is given as follows.
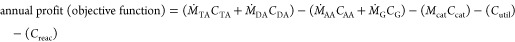
where *Ṁ* is the annual mass produced (kg/year), *M* is the annual demand (kg/year), *C* is the market value ($/kg), *C*_util_ is the annual cost of utility ($), and *C*_reac_ is the cost of the reactor ($); TA is triacetin; DA is diacetin; AA is acetic acid; G is glycerol; cat is catalyst.

## 3 Techno-Economic Analysis

Following flowsheet optimization, a techno-economic analysis was conducted to quantitatively assess the commercial viability of the process, deploying the novel catalyst, Sn-DTP/K-10 as used in our recent study, based on a 20-year plant operating life with a 1 year start-up.^36^ When considering the annual cost of the catalyst, it was estimated that the catalyst would require replacement every 3 months, a commonly employed approximation. As is customary with economic assessments associated with plant development and operation, the overall costs may be distinctly categorized into two distinct classes, namely, capital costs and manufacturing costs.^38^ The economic analysis assumes that the profit remains constant for the project lifetime and that the product is sold immediately after the first batch. Also, it has been assumed that the production begins right from day 1 of the project. The following section of the report aims to outline the methodology adopted in providing accurate estimations of such costs, utilizing approaches commonly adopted within the literature.

### 3.1 Estimation of Capital Costs

**Figure 8:**
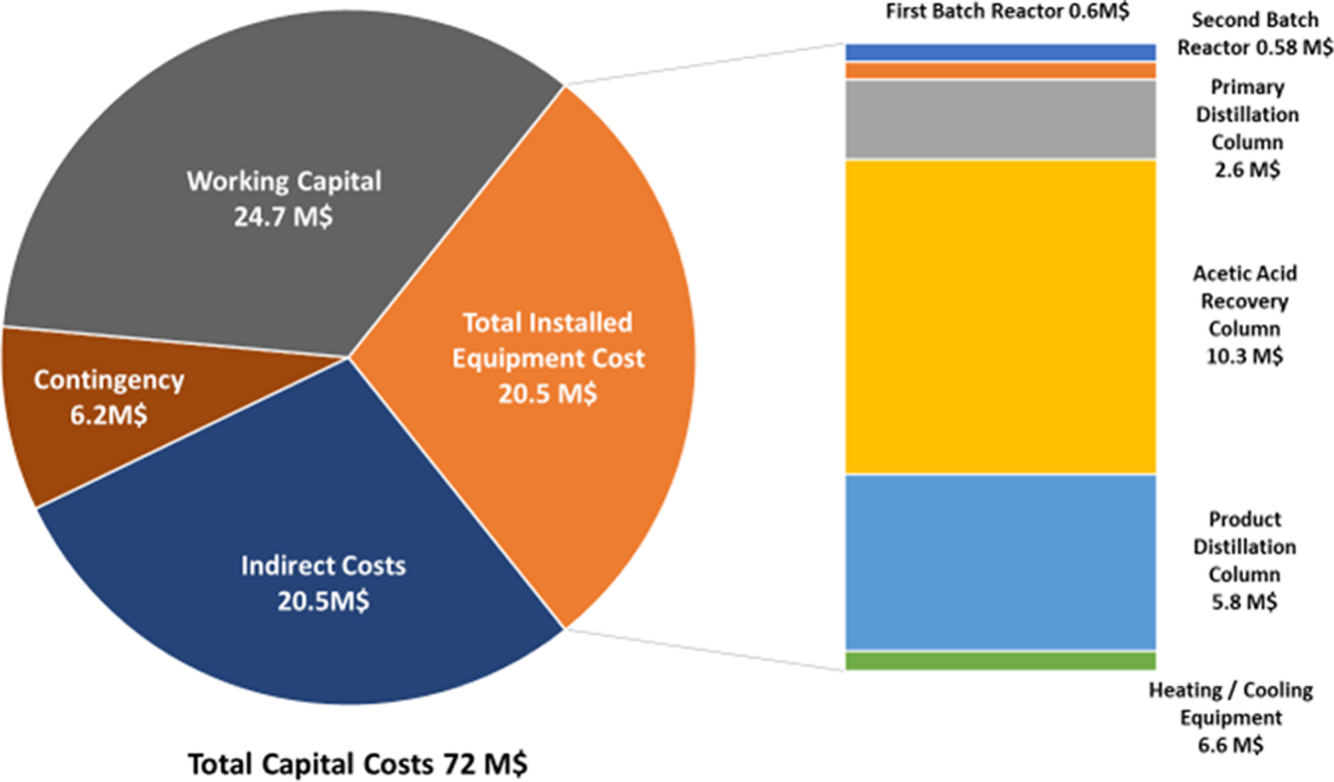
Breakdown of capital costs.

The total capital costs for the project are estimated to be 72 M$. The total capital cost has been calculated as a sum of process equipment costs, indirect costs that include installation and auxiliary facilities costs, contingency costs, and the working capital costs. The fixed capital costs of process equipment were estimated using Aspen’s built-in capital cost estimator (ACCE), which includes the bulk costs of equipment, any additional instrumentation likely to be required, and labor costs for installation. The indirect costs as mentioned by Turton et al. can be in the range of 20–100% of the installed equipment costs.^39^ For the purpose of this study, a conservative estimate of 100% has been set of indirect costs. The contingency costs were estimated to be 15% of the fixed capital as recommended by Turton et al.^39^ Finally, the working capital was estimated as one month’s operating expenses, without considering equipment and capital-related costs. Similarly, this is a common approximation utilized within the literature,^38,40,41^ with analogous approximation methods reported by Garrett^42^ and Towler and Sinnott.^43^ A detailed breakdown of the capital costs can be seen in Figure 8.

### 3.2 Estimation of Operating Costs

The operating costs can be further subdivided into mainly direct variable costs, fixed costs, and general expenses. The annual raw material costs and catalyst demand were computed through the known required annual masses, attained following the flowsheet optimization procedure, and their known market prices. The market prices of the products and reactants are mentioned in Table 3. The cost of maintenance and repairs was estimated as 10% of the fixed capital investment, as recommended by Apostolakou et al.^44^ Utility costs were similarly estimated directly by Aspen Plus, with the cost of waste estimated using the correlation factor reported by Ulrich and Vasudevan.^45^ Considering that industrial batch manufacturing of this scale is largely automated to ensure a high level of processing control, there is no requirement for a significant workforce.^38^ Consequently, it was approximated based on the proposed plant capacity, and seven operators are required per shift.^41^ As recommended by Turton et al.^39^ through multiplication of this number by 4.5, the total number of operators may be computed, with such a factor taking into consideration that an average operator works 49 weeks a year and completes five 8 h shifts per week. The average salary of an operator was approximated as $57,500/annum, based on the average annual EU salary for such a role.^46^ Supervisory and clerical wages were approximated as 20% of the total annual operator costs, as adopted by Haas et al.^41^ The rate of annual depreciation was computed utilizing the straight-line depreciation method, considering a plant life of 20 years and a zero-salvage value. Estimations based on plant overhead costs, interest, taxes, and insurance were predicted utilizing correlations reported by Martinovic et al.^38^ General expenses refer to costs associated with the distribution and selling of the product, as well as administrative costs; distribution costs were estimated as 3% of the total manufacturing cost, with administrative costs approximated as 15% of the sum of the direct labor and maintenance and repairs costs. The approach adopted above is analogous to that recently reported by Martinovic et al.^38^ when comparing the economic viability of alternative biodiesel processing strategies and thus may be considered a viable methodology.

**Table 3:** Prices of Reactants and Products

compound	cost ($/kg)
glycerol	0.4
acetic acid	1.2
catalyst	0.47
diacetin	1
triacetin	2

**Figure 9:**
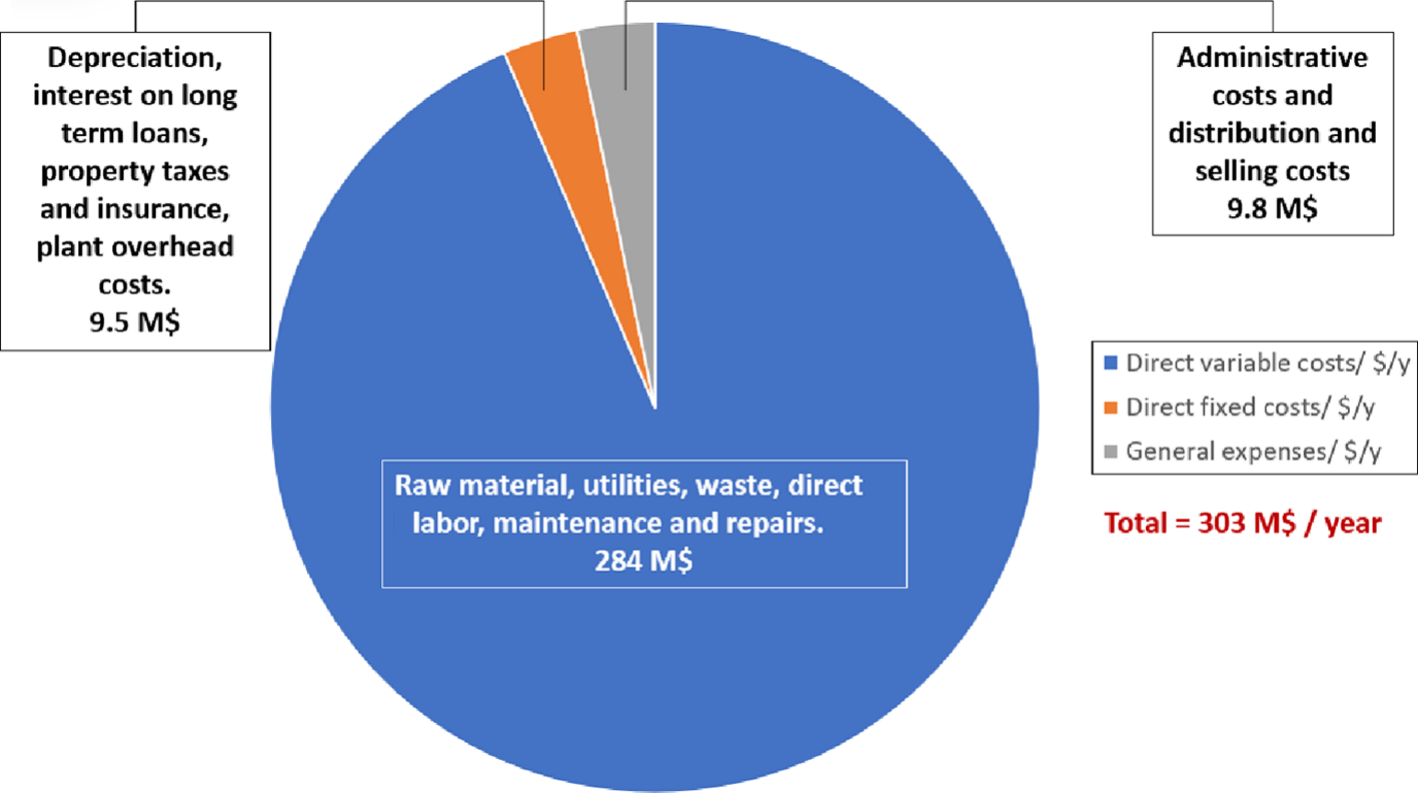
Breakdown of manufacturing costs/year.

Figure 9 shows the breakdown of the manufacturing costs associated to the project.

### 3.3 Economic Performance Indicators

To provide a further indication of the economic viability of the process, the net present value (NPV) of the process was determined. A discounted cash flow approach was used for the accurate estimation of the NPV of the project. As such, it is the predominant economic indicator utilized within the majority of techno-economic analysis studies.^9,47^ The discounted rate was set as 15.3%, as adopted recently by Al-Saadi et al.^9^Table 4 shows the key findings of the techno-economic survey for the optimized two-stage process.

**Table 4:** Economic Evaluation of the Optimized Two-Stage Process Based on a Glycerol Capacity of 10,000 Tons/Year

total capital investment (M$)	71
total manufacturing cost (M$/year)	303
annual gross profit (M$/year) including corporation tax of 19%	60.5
NPV at 20 years (M$)	235
payback period (years)	1.7

**Figure 10:**
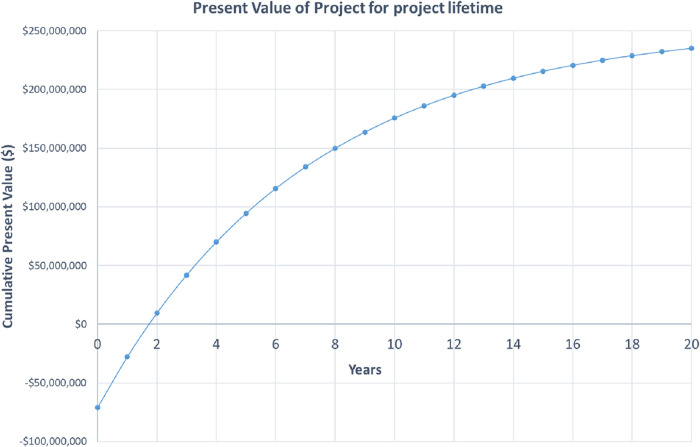
Present value of the project for project lifetime.

Figure 10 shows the cumulative present value of the project for the project lifetime of 20 years. Based on the figure, the payback period where the NPV is zero is at 1.7 years.

### 3.4 Estimation of the Minimum Selling Price

The current selling price of the biodiesel additive product based on a typical biofuel additive product (∼0.86 wt % TA, ∼0.14 wt % DA) was estimated to be 1.586 $/kg. This was based on the current market values of TA and DA. To estimate the minimum selling price (MSP), the NPV was set to 0, and the minimum selling price of the product was estimated to be 1.38 $/kg. However, it must be noted that the current market price and trend are well above the MSP. This approach was used by Yang and Rosentrater^47^ for estimation of the MSP of bioadhesive.

### 3.5 Sensitivity Analysis

**Figure 11:**
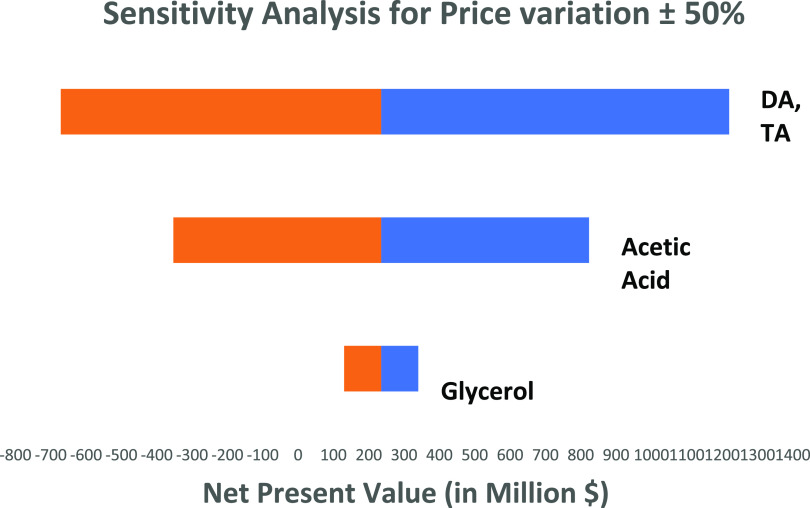
Sensitivity analysis on the effect of NPV.

To assess the impact of market price volatility on the economic viability of the proposed process, a sensitivity analysis procedure was conducted whereby the defined current market price was fluctuated by ±50%, an approach recently adopted by Al-Saadi et al.,^9^ investigating an alternative glycerol valorization process. Figure 11 shows the sensitivity analysis and its effect on the NPV.

The sensitivity analysis indicates that the project is highly sensitive to the price of the products, which are diacetin and triacetin, and a major fluctuation in the price can render the project to be unfeasible. However, due to the rising market and growing market trends for such products, it seems very unlikely.^48^ Furthermore, a fluctuation in acetic acid price beyond a 30% increase can take the NPV to 0. A change in the crude glycerol price can make the project more profitable, and due to the increased volume of production of crude glycerol, the price of crude glycerol is set to decrease based on the current market trends.^49^

## 4 Future Scope and Conclusions

This study analyzes the economic feasibility of a two-stage process for the production of biofuel additives like diacetin and triacetin from crude glycerol with acetic acid as the reactant and Sn-DTP/K-10 as the catalyst. The findings of the economic analysis revealed the process to be highly profitable and thus definitively confirmed the commercial viability of the novel catalyst at an industrial manufacturing scale. The economic analysis has shown that the project is highly profitable with an NPV of 235 M$ for a project lifetime of 20 years. As shown by the sensitivity analysis, the project is stable as there are no major price changes predicted in the near future. Future studies can be aimed at a comparative analysis between the two- and three-stage production process for a better economic understanding. Alternative continuous production routes can also be explored.

## Notes

For the purpose of open access, the author has applied a Creative Commons Attribution (CC BY) license to any Author Accepted Manuscript version arising.
